# The Effects of High-volume Hemofiltration by Different Ultrasound Directing on Extra Vascular Lung Water Index in Patients with Septic Shock

**Published:** 2018-09

**Authors:** Hongsheng REN, Bo SONG, Pengcheng LI, Cheng HUAN, Yufeng CHU, Min DING, Yuping WANG, Qingchun YAO, Peng WANG, Guoqiang QI, Chunting WANG

**Affiliations:** 1. Dept. of Intensive Care Unit, The Provincial Hospital Affiliated to Shandong University, Jinan, China; 2. Dept. of Intensive Care Unit, Taishan Medical University Affiliated Zouping Hospital, Zouping, China

**Keywords:** Critical bedside ultrasound, Septic shock, High-volume hemofiltration

## Abstract

**Background:**

We explored the effects of high-volume hemofiltration(HVHF) by different ultrasound directing on the plasma N-terminal pro-B-type natriuretic peptide(NT-Pro-BNP), extra vascular lung water index (EVLWI), liquid net balance quantity and prognosis in patients with septic shock.

**Methods:**

Overall, 107 intensive patients with septic shock were enrolled by retrospective analysis from Department of Intensive Care Unit (ICU) of the Shandong Provincial Hospital affiliated to Shandong University from 2014–2017. According to HVHF by different ultrasound directing, all the patients were divided into two groups ((ultrasonic cardiac output monitor (USCOM), group A, n=51cases)) and ((critical bedside ultrasound (CBU), group B, n=56cases)).

**Results:**

The value of CI in group A had a significant positive correlation with the value of PCCI by the PiCCO_2_ monitoring (*P*<0.05). The lung ultrasound water B lines in group B also had a significant positive correlation with the value of EVLWI by the PiCCO_2_ monitoring. The cumulative liquid net balance quantity in group B had a more significant elevation than group A after treatment 7th d. The level of EVLWI after treatment 48 h and 72 h, the level of plasma NT-Pro-BNP, the levels of P(A-a)DO2,OI and blood lactic after treatment 72 h, and the APACHE II scores and SOFA scores after treatment 7thd were reduced more significantly in group B than group A (*P*<0.001). The mortality at 28th day had a more significant decrease in group B than group A.

**Conclusion:**

It could decrease the level of NT-Pro-BNP, EVLWI, P(A-a)DO2, which then improves pulmonary oxygenation. Consequently, it decreased the APACHE II and SOFA scores and improved the 28th survival rate of patients.

## Introduction

The early goal-directed therapy is hardly to prevent the progress of septic shock. Its death rate is staying at high level. Serious pyohemia and infection shock guideline (2012) showed mortality in septic shock was 20%–40% (1). Even though fluid resuscitation is one of the main methods to treat septic shock, fluid overload and pneumonedema caused by inappropriate fluid resuscitation can influence the prognosis of patients (2). It is difficult to recognize fluid overload in the early time. The symptoms, physical signs and laboratory detections which used to judge fluid overload is atypical and belate. The sensitivities that had used beside x-ray chest judge pneumonedema is very low. Now pulse indicator contour cardiac output (PiCCO_2_) and extra vascular lung water index (EVLWI) is the common objective assessments of pulmonary edema (3). But it is costly, invasive and more complications. It also cannot be with continuous use.

There are no effective and non-invasive monitoring methods for pneumonedema. Beside ultra-sound starts to be used in severe patients (4). During the progress of our retrospective analysis which compared different lung ultrasound and PiCCO_2_ monitoring, guided the amount of liquid recover under the high volume hemo-filtration (HVHF), developed appropriate liquid management strategies via the changes of acute physiology and chronic health evaluation-II (APACHEII), sepsis-related organ failure assessment (SOFA). Our aim was to reduce the fatality rate of septic shock.

## Material and Methods

### Patients’ data

We retrospectively analysed 107 patients who admitted to our hospital’s Department of Critical Care Medicine (ICU) from 2014–2017. The patients were diagnosed as septic shock, took capacity recovery, PiCCO_2_ monitoring and high-volume hemofiltration (HVHF). HVHF was implemented at least 72 h.

Inclusion criteria: Diagnose of all patients according with the diagnostic criteria of septic shock (5), age≥18 yr old, age ≤ 80 yr old. According to its implement ultrasonic cardiac output monitoring (USCOM) and critical bedside ultrasound (CBU) monitor, all of patients were divide into two groups. Group USCOM (group A), n=51 cases, male 28, female 23, age 22–78 yr old, average (52.7±15.6) yr old; Group CBU (group B) n=56 cases, male 32, female 24, age 24–79 yr old, average (54.3±16.2) yr old. There was no significant difference between the two groups in age gender disease severity according to APACHE II and SOFA scores.

Exclusion criteria: HIV infected cases, advanced cancer patients, end-of-life patients, patients with cardiogenic shock, pregnant woman with obstructive shock, patients with leukemia or who refused to participate.

Removed criteria: the patients who failed to complete the 28-day survival rate of follow-up, gave up treatment halfway, or went to other hospital for treatment.

This study met medical ethical standards with the approving of the Hospital Ethics Committees of Shandong provincial. Written informed consents were obtained from all subjects.

## Methods

All patients were monitored immediately and at 24 h, 48 h, 72 h after treatment. We extracted the patient’s blood, and then tested the patient’s blood routine, coagulation, liver and kidney faction. We took 1.8 ml of venous blood to 1.09 mol/L sodium at rate 9:1 anticoagulation, 3000 r/min 15minutes centrifuge, serum −20 °C preservation. The immunochromatographic method was used to determinate PCT goal by standard diagnostic kit (BRAHMS Company, Germany). RocheElecys 2010 electrochemiluminescence immunoassay analyser was used to determinate NT-proBNP by NT-pro BNP kits (Roche Company, Switzerland). All operations were inferred strictly accordance with the instruction.

We recorded the difference between the amount of input and output of 24 h and calculated net balance of accumulated fluid for seven days (6). From the patient right internal jugular vein or right subclavian vein, we inserted into the central venous catcher, connected PiCCO_2_ temperature probe. From patients’ arteria femoral, we inserted into PiCCO_2_ catheter (PV2015L20, PULSION Company, Germany). We connected to the monitor which had PiCCO_2_ module. We monitored patient’s PCCI, EVLWI continuously. We rapidly injected 15 ml of ice salme from the temperature probe which was connected with the intravenous or subclavian venous catchers. All surveys were measured at least three times in a row. We took the average of three valid measurements as each monitoring data (7).

We had done consciously beside high-flow venous hemofiltration at least 72 h. We used femoral vein indwelling catheter, high-volume continuous, pre-replacement method 70% or completely after the replacement, changed the filter each 24 h, the anticoagulation was heparin or sodium citrate. The amount of replacement liquid was 3000–4000 ml/h (50–70 ml·kg^−1^·h^−1^), the amount of replacement was beyond 60 L/d, the blood flow was 150∼200 ml/min (8). According to patient’s different dates under the guidance of ultraphonic, we adjusted ultrafiltration volume.

### USCOM monitor

We used the continuous ultrasound noniasive cardiac output detector of USCOM (company, Australia) (9) to get the best blood flow chart and data. We asked the patient to lie in the supine position, put the probe on the suprasternal fossa and increased the pressure appropriately until a triangular systolic flow signal spectrogram appeared. All surveys were measured three times and we took the average.

### CBU monitor

We used the portable ultrasound machine for monitoring, phased array (1–5 MHZ) probe. The radical bundle extending from pleural line to route and decaying with the breathing changes was B line. According to pulmonary ultrasound guide recommendation, we selected eight sections of the front chest wall to observe lung water. If more than three lines appeared in an inter-costal space section, then we believed the B line was positive (10).

### Condition score

At the beginning, 72 h, 7 d after treatment, we conducted APACHE-II and SOFA scores (11,12) and counted the survival and deaths of patients of 28 d followed-up.

### Statistical method

All the measurement data were expressed as mean ± standard deviation, *t*-test between two groups, the incidence with x^2^ test, correlation analysis using Person correlation. All the data were processed by SPSS 17.0 computer software (Chicago, IL, USA). If the *P* value was <0.05, we regarded the difference was statistical significance.

## Results

### Correlation analysis

The cardiac output index (CI) in group A was significant positively correlated with the continuous cardiac output index (PCCI) monitored by PiCCO_2_ (correlation coefficient R=0.689, 95% confidence interval (0.607, 0.769), 0.01<*P*< 0.05. There was also a significant positive correlation between the number of pulmonary ultrasound B in group B and the extravascular lung water index (EVLWI) monitored by PiCCO_2_ (correlation coefficient R = 0.705, 95% confidence interval (0.657, 0.815), 0.01 <*P*< 0.05.

### Change of cumulative liquid net balance quantity

There was no significant difference in the net balance of liquid between the two groups at 24 h, 48 h, 72 h after treatment. After 7th d, the net balance of accumulated liquid in group B was significant different from that in group A (1276 ± 248 vs 537 ± 39, *t* = 21.04, *P* = 0.001).

Before treatment, after treatment 24 h, 48 h, 72 h, there was no significant difference in plasma PCT level between group B and group A Before treatment, after treatment 24 h, 48 h, the level of plasma of NT--Pro BNP had no significant change, but after treatment of 72 h, the level of plasma NT-Pro BNP decreased, and it was statistically significant (Table 1).

**Table 1:** Comparison of inflammatory cytokines and cardiac function parameter in serum level between groups as mean ± standard deviation

***Group***	***Number***	***PCT (ug/L)***				***NT-Pro BNP (ug/L)***			
		***0h***	***24h***	***48h***	***72h***	***0h***	***24h***	***48h***	***72h***
Group A	51	49.3±15.2	31.7±12.3	18.2±8.1	8.3±3.2	2975.8±198.4	1868.5±138.5	1265.2±90.7	804.9±56.2
Group B	56	47.2±12.5	32.4±11.2	17.3±8.2	7.1±2.9	2909.4±188.3	1825.4±145.9	1237.5±86.7	721.8±43.9^*^
		*t*=0.776	*t*=0.321	*t*=571	*t*=1.616	*t*=1.866	*t*=1.567	*t*=1.611	*t*=8.466
		*P*=0.415	*P*=0.625	*P*=0.509	*P*=0.125	*P*=0.065	*P*=0.142	*P*=0.135	*P*=0.001

Before treatment, after treatment 24 h, 48 h, 72 h, thoracic blood volume index (ITBVI) and global end-diastolic volume index (GEDVI) had no significant difference in two groups. There was no significant difference between the two groups in PVPI and EVLWI before treatment, 24 h, 48 h after treatment, but the pulmonary vascular permeability in group B was significantly higher than that in group A (PVPI) and extravascular lung water index (EVLWI) decreased, they were statistically significant (Table 2).

**Table 2:** Comparison of hemodynamic parameters level between groups (mean ± standard deviation)

**Group**	**Number**	**ITBVI (ml/m^2^)**				**GEDVI (ml/m^2^)**			
		***0h***	***24h***	***48h***	***72h***	***0h***	***24h***	***48h***	***72h***
Group A	51	1304.2±373.5	1163.4±368.0	1079.4±295.5	967.8±242.9	1259.7±415.2	1258.2±372.3	1263.3±351.0	1079.4±285.2
Group B	56	1265.6±363.7	1244.5±327.4	1141.6±269.3	901.2±204.5	1391.6±404.6	1175.4±302.3	1164.2±295.8	995.9±294.3
		*t*=1.229	*t*=1.199	*t*=1.134	*t*=1.528	*t*=1.662	*t*=1.255	*t*=1.571	*t*=1.489
		*P*=0.239	*P*=0.182	*P*=0.263	*P*=0.137	*P*=0.092	*P*=0.252	*P*=0.145	*P*=0.163

**Group**	**Number**	**PVPI**				**EVLWI (ml/kg)**			
		***0h***	***24h***	***48h***	***72h***	***0h***	***24h***	***48h***	***72h***
GroupA	51	13.4±4.5	12.3±3.5	11.4±3.2	10.8±3.5	19.5±6.3	17.2±5.2	13.8±4.7	11.5±3.3
GroupB	56	12.9±3.5	11.9±2.9	9.8±3.4^*^	6.1±2.7^**^	18.9±5.6	15.4±5.0	11.7±4.2^*^	7.3±3.1^**^
		*t*=0.637	*t*=0.641	*t*=2.507	*t*=7.723	*t*=0.519	*t*=1.821	*t*=2.428	*t*=6.768
		*P*=0.699	*P*=0.629	*P*=0.029	*P*=0.001	*P*=0.742	*P*=0.082	*P*=0.035	*P*=0.001

Compared with group A,

* *P*<0.05,

** *P*<0.01

The difference between the alveolar oxygen pressure and the partial pressure of arterial oxygen (P(A-a)DO2), oxygenation index (OI) and arterial lactate (Lac) in the two groups were not significant before and 24 h and 48 h after treatment.

However, at 72 h after treatment, the difference between alveolar oxygen pressure and arterial oxygen pressure (P(A-a)DO2), oxygenation index (OI) and arterial lactate (Lac) had decreased significantly, it was statistically significant (Table 3). There was no significant difference in APACHE-**II** score and SOFA score between the two groups before treatment and at 72 h, however, APACHE-**II** scores and SOFA scores in group B were significantly lower than those in group A at 7th day after treatment, both of which had a statistically significant meaning (Table 4).

**Table 3:** Comparison of Alveolar-arterial oxygen pressure difference P(A-a) DO2, oxygenation index (OI) and artery atrial blood lactic between two groups (mean ± standard deviation)

***Group***	***N***	***P(A-a) DO2 (mmHg)***				***Oxygenation index OI***				***LAC (mmol/l)***			
		***0***	***24h***	***48h***	***72h***	***0***	***24h***	***48h***	***72h***	***0***	***24h***	***48h***	***72h***
Grop A	51	315.7±47.7	290.5±46.5	250.2±38.7	221.2±29.3	112.4±34.2	127.7±41.5	131.8±37.3	158.2±33.4	6.3±2.4	4.6±2.0	3.6±2.3	3.1±1.7
Grop B	56	305.4±52.6	276.2±48.3	236.7±39.5	181.9±21.8^**^	124.5±36.6	138.3±30.7	140.4±31.2	198.7±41.5^**^	6.7±2.1	3.8±2.1	2.9±1.3	1.8±0.7^**^
		*t*=1.062	*t*=1.559	*t*=1.785	*t*=7.780	*t*=1.768	*t*=1.491	*t*=1.287	*t*=5.583	*t*=0.914	*t*=1.766	*t*=913	*t*=5.082
		*P*=0.323	*P*=0.184	*P*=0.078	*P*=0.001	*P*=0.082	*P*=0.139	*P*=0.235	*P*=0.001	*P*=0.394	*P*=0.089	*P*=0.073	*P*=0.001

Compared with group A,

* *P*<0.05,

** *P*<0.01

**Table 4:** Comparison of acute physiology and chronic health evaluation **II** (APACHE **II**), sepsis-related organ failure assessment (SOFA) after high-volume hemofiltration (HVHF) between groups (mean ± standard deviation)

***Group***	***Number***	***APACHE II***			***SOFA***		
		***0***	***3d***	***7d***	***0***	***3d***	***7d***
Group A	51	24.5±8.3	19.4±8.2	14.3±6.5	15.9±6.1	13.9±4.8	12.8±3.8
Group B	56	25.1±7.4	17.3±7.1	8.2±3.3^**^	16.5±5.7	12.6±3.3	8.2±3.1^**^
		*t*=0.393	*t*=1.409	*t*=7.175	*t*=0.524	*t*=617	*t*=6.822
		*P*=0.637	*P*=0.178	*P*=0.001	*P*=0.495	*P*=0.132	*P*=0.001

Compared with group A,

* *P*<0.05,

** *P*<0.01

After 28 d of following-up, there were 17 dead patients in group A and 9 dead patients in group B, it had significant differences in mortality rates (33.33% vs 16.07%, X^2^ = 4.324, *P* <0.05).

## Discussion

When the body is in systemic inflammatory response syndrome (SIRS), severe sepsis, or septic shock, the plasma inflammatory mediators will increase damage capillary endothelial cells then increase pulmonary vascular permeability (PVP), cause extra-vascular lung water index (EVLW). These changes will decrease Alveolar-arterial oxygen exchange capacity and lead to tissue hypoxia-arterial blood lactate increase, as result, the patients would develop a multiple organ dysfunction syndrome (13).

Research showed that normal human PCT concentration is 0.033 ug/L. As a biomarker, serum PTC level has a high degree of specificity and sensitivity which can be used to monitor bacterial infections. This can reflect the severity of the disease and evaluate the patient’s prognosis (14, 15). Ventricular myocytes produce NT-proBNP by the increased stimulation of left ventricular walls pressure. NT-proBNP is the predictors of left ventricular preload and cardiac function. According to reports (16), NT-proBNP can be used as the marker to recognize sepsis-induced heart failure. NT-pro BNP reflects the preload of heart which is positively correlated with global enddiastolic volume index (GEDVI) and intrathoracic blood volume index (ITBVI).

PiCCO_2_ has become the clinical gold standard for hemodynamic monitoring of patients and been used to guide the treatment shock. Research confirmed the accuracy of PiCCO_2_. Using the technology of PiCCO_2_ to measure PCCI which can reflect heart pumping blood faction, EVLWI which can reflect the severity of pulmonary directly. All of this are closely related to the prognosis of critically ill patients (17). It had showed that noninvasive and repeatable cardiac output monitoring cardiac index (CI) such as USCOM, which reflected the change of heart pumping blood (9). Slight lung hydrostatic pressure increase can cause B line. Ultrasonic lung water monitoring can reflect the dynamic change of body capacity which can help guide the more individual and accurate capacity management. As an index, pulmonary ultrasound B line can be used to reflect the condition of pneumonedema, it had been proved by a research (18–20). The total number of ultrasound B-lines and EVLW has a good correlation for lung oxygenation function and acute respiratory distress syndrome (ARDS) patients (21). Our research showed that there had a significant positive correlation between the CI and the PCCI monitored by PiCCO_2_ in group A. (Correlation coefficient R = 0.689). There was also a significant positive correlation between the number of pulmonary ultra-sound B line and EVLWI monitored by PiCCO_2_ (Correlation coefficient R = 0.705).

Early use of PiCCO_2_ hemodynamic monitory was related to non-invasive use USCOM monitor cardiac output index. There are good correlations between pulmonary ultrasound B line and PiCCO_2_. The USCOM and the CBU have the advantages of non-invasive and can be repeated. More importantly, it is that pulmonary ultrasound can play the advantages of non-invasive, simple and economical, glide to capacity management better. Our research showed that two groups of 24, 48, 72 h after treatment, there was no significant difference of liquid between the two groups. There was significant different of accumulate liquid net balance between the two groups amount when 7 d after treatment.

NT-proBNP is caused by excessive pulling off ventricular cells when the capacity is too heavy. At that time, there is already a serious overload or even with heart failure. For critical ill patients, NT-proBNPcan not predict the increase of cycle capacity and heart failure. As a monitoring indicator GEDVI and ITBVI, that can reflect the pre-load of heart and heart failure. They can reflect the information of capacity accurately which more sensitive than NT-proBNP. EVLWI reflect directly pulmonary edema and its severity, provide evidence for finding pulmonary edema timely and accurately. It is more responsive than GEDVI and ITBVI, in reflecting early capacity overload and heart failure (19). Monitoring PVPI, EVLWI can evaluate capillary permeability capacity load, heart failure and high EVLWI patients have poor prognosis, they need longer mechanical ventilation. Our research showed that PCT had no significant difference before treatment, after treatment 24, 48, 72 h between two groups. But the plasma NT-proBNP concentrations in group B were lower than those in group after treatment 72 h. The difference was statically significant. There was no difference between this two groups in ITBVI, GEDVI, PVPI, EVLWI before treatment and after treatment 24 h, 48 h. There was significant difference between two groups in EVLWI after treatment 72 h.

During the process of fluid resuscitation of septic shock, pulmonary capillary leak syndromeis the most common complication. It can lead to reduced ARDS. It is crucial to recognize ARDS. We should considerate (P(A-a)DO2), OI, Lac which can evaluate early lung oxygenation function (21). Out research showed that there was no significant difference between the two groups in (P(A-a)DO2), OI,Lac before treatment and after treatment 24, 48 h. It was statistically significant after treatment 72 h.

Research confirmed that pulmonary ultrasound B-line monitoring was more accurate and sensitive to judge the increase and elimination of the pulmonary water in patients with advanced disease sensitively and accurately. Pulmonary ultra-sound B line is more sensitive and accurate than NT-proBNP. Our research showed that B line which was displayed by ultrasound can guide high-volume filtration (HVHF) to decrease better EVLWI. It can effectively prevent the occurrence of extravascular lung water, reduce inflammatory mediators and pulmonary capillary permeability. The patients in insufficient effective circulation need full and timely recovery of capacity to maintain a stable cycle. Early goal-directed therapy (EGDT) can effectively improve patient survival rate, but excess fluid load can make the patient’s prognosis worse (22). Cordemans propose a concept of Late Goal Directed after Fluid Resuscitation (LGDFR) (23). Malbrain propose a precept of ROSE (24). However, there are no specific research data to tell when the LGDFR begins, finish and the amount of grasped dehydration.

Open fluid recovery and liquid overload can lead increasing of inducing the needs of organ support, mechanical ventilation time and complication, even increases fatality rate. New England Journal of Medicine published the outcome proCESS, it leads larger clinical controversy (25). Compared with the general treatment EGDT, it does not reduce fatality rates, hospitalization time, the time of living in ICU better. It also does not reduce organ support time (26).

Circulatory capacity overload seriously affects the outcome of patients. It is necessary to strengthen supervision and do goal-oriented dehydration therapy because the fight against patients is relatively hidden. We should have aggressive volume recovery early to correcting low perfusion. We can achieve the best condition through titration capacity management (27). When the condition of patient improves gradually, reduce the support and dehydration to reduce excess circulation capacity. Effective clearance capacity after resuscitation volume overload is beneficial to patient prognosis. Gordmans found that it could reduce extravascular lung water and improve patient’s prognosis by PEEP and using albumin furosemide for dehydration (28). Our research accurately adjusted the capacity by using high-volume hemofiltration (HVHF), achieved refinement of capacity recovery and management strategy by critical ultrasound directing.

Ensuring organ perfusion and avoiding fluid overload is the essential to the treatment of septic shock. Therefore, objective, effective, especially non-invasive monitoring methods are necessary. After early full capacity of recovery, we should properly limit lipid intake properly and timely, then get into the late dehydration. For patients with poor cardiac function, we should pay more attention to capacity overload, closely monitor and implement late capacity management. Pulmonary ultrasound B line can reflect the capacity of critically ill patients accurately and sensitively. Our research show that: after treatment 7 d, the scores of APACHE-II, SOFA in group B were significantly lower than A group. The difference was statistically significant. After 28 d of following, the mortality rate had significant difference between the two groups.

## Conclusion

For patients with septic shock, it is more effective depending on lung water B line monitored by pulmonary ultrasound than on heart output monitored by USCOM. The former can better guide HVHF to control liquid negative balance, decrease the level of plasma NT-proBNP, reduce extravascular lung water index and the difference in alveolar-arterial oxygen exchange. It can also better improve oxygenation, lower the APACHE-**II**’s rating and SOFA’s rating, improve patient’s outcomes after 28 d. However, this research was a retrospective study so that we should use prospective randomized study in the further studying.

## Ethical considerations

Ethical issues (Including plagiarism, informed consent, misconduct, data fabrication and/or falsification, double publication and/or submission, redundancy, etc.) have been completely observed by the authors.

## References

[1] León AL Hoyos NA Barrera LI (2013). Clinical course of sepsis, severe sepsis, and septic shock in a cohort of infected patients from ten Colombian hospitals. BMC Infect Dis, 13: 345–347. 23883312 10.1186/1471-2334-13-345 PMC3727953

[2] Bouchard J Mehta RL (2010). Fluid balance issues in the critically ill patient. Contrib Nephrol, 164: 69–78. 20427995 10.1159/000313722

[3] Litton E Morgan M (2012). The PiCCO2 monitor: a review. Anaesth Intensive Care, 40:393–409. 22577904 10.1177/0310057X1204000304

[4] Baldi G Gargani L Abramo A (2013). Lung water assessment by lung ultrasonography in intensive care: a pilot study. Intensive Care Med, 39:74–84. 23052950 10.1007/s00134-012-2694-x

[5] Dellinger RP Levy MM Rhodes A (2013). Surviving sepsis campaign: international guidelines for management of severe sepsis and septic shock, 2012. Intensive Care Med, 39(2):165–228. 23361625 10.1007/s00134-012-2769-8 PMC7095153

[6] Yao B Liu DW Wang XT Zhang HM (2014). Negative fluid balance predicts survival in patients with septic shock. Zhonghua Yi Xue Za Zhi, 91: 3206–3210 [In Chinese]. 25604218

[7] Lu NF Zheng RQ Lin H Shao J Yu J (2014). Clinical studies of surviving sepsis bundles according to PiCCO on septic shock patients. Zhonghua Wei Zhong Bing Ji Jiu Yi Xue, 26:23–27 [In Chinese]. 24506852 10.3760/cma.j.issn.2095-4352.2014.01.005

[8] Chen XF Ye JL Zhu ZY Xue H Pu XH Miao XL (2014). Evaluation of high volume hemofiltration according to pulse-indicated continuous cardiac output on patients with acute respiratory distress syndrome. Zhonghua Wei Zhong Bing Ji Jiu Yi Xue, 26:650–654 [In Chinese]. 25230867 10.3760/cma.j.issn.2095-4352.2014.09.009

[9] Chong SW Peyton PJ (2012). A meta-analysis of the accuracy and precision of the ultrasonic cardiac output monitor(USCOM). Anaesthesia, 67:1266–1271. 22928650 10.1111/j.1365-2044.2012.07311.x

[10] Volpicelli G Elbarbary M Blaivas M (2012). International evidence-based recommendations for point-of-care lung ultarsound. Intensive Care Med, 38: 577–591. 22392031 10.1007/s00134-012-2513-4

[11] Adam F1 Bor C Uyar M Demırağ K Çankayalı İ (2013). Severe acute pancreatitis admitted to intensive care unit:SOFA is superior to Ranson’s criteria and APACHE II in determining prognosis. Turk J Gastroenterol, 24:430–435. 24557967 10.4318/tjg.2013.0761

[12] Qiao Q Lu G Li M Shen Y Xu D (2012). Prediction of outcome in critically ill elderly patients using APACHE II and SOFA scores. J Int Med Res, 40: 1114–1121. 22906285 10.1177/147323001204000331

[13] Kim YA Ha EJ Jhang WK Park SJ (2013). Early blood lactate area as a prognostic marker in pediatric septic shock. Intensive Care Med, 39: 1818–1823. 23818093 10.1007/s00134-013-2959-z

[14] Layios N Lambermont B (2013). Procalcitonin for antibiotic treatment in intensive care unit patients. Curr Infect Dis Rep, 15: 394–399. 23933824 10.1007/s11908-013-0360-2

[15] Suberviola B1 Castellanos-Ortega A González-Castro A García-Astudillo LA Fernández-Miret B (2012). Prognostic value of procalcitonin, C-reactive protein and leukocytes in septic shock. Med Intensiva, 36: 177–184 [In Spanish]. 22055776 10.1016/j.medin.2011.09.008

[16] Takahashi EA1 Moran SE Hayashi MS Inouye DS Takanishi DM Jr Yu M (2013). Brain-type natriuretic peptide and right ventricular end-diastolic volume index measurements are imprecise estimates of circulating blood volume in critically in subjects. J Trauma Acute Care Surg, 75: 813–818. 24158199 10.1097/TA.0b013e3182a85f3a

[17] Jozwiak M Silva S Persichini R Anguel N Osman D Richard C Teboul JL Monnet X (2013). Extravascular lung water is an independent prognostic factor in patients with acute respiratory distress syndrome. Crit Care Med, 41: 472–480. 23263578 10.1097/CCM.0b013e31826ab377

[18] Enghard P Rademacher S Nee J Hasper D Engert U Jörres A Kruse JM (2015). Simplified lung ultrasound protocol shows excellent prediction of extravascular lung water in ventilated intensive care patient. Crit Care, 19: 36. 25656060 10.1186/s13054-015-0756-5 PMC4335373

[19] Trezzi M Torzillo D Ceriani E (2013). Lung ultrasonography for the assessment of rapid extravascular water variation: evidence from hemodialysis patients. Intern Emerg Med, 8: 409–415. 21590437 10.1007/s11739-011-0625-4

[20] Bataille B Rao G Cocquet P Mora M Masson B Ginot J Silva S Moussot PE (2015). Accuracy of ulcrasound B-line score and E/Ea ratio to estimate extravascular lung water and its variations in patients with acute respiratory distress syndrome. J Clin Monit Comput, 29:169–76. 24819560 10.1007/s10877-014-9582-6

[21] Sun LX Gao XJ Li ZB Feng QS Wang ZY Wang WJ Xu L (2014). The prognostic value of extravascular lung water index in patients with acute respiratory distress syndrome. Zhonghua Wei Zhong Bing Ji Jiu Yi Xue, 26: 101–105 [In Chinese]. 24524400 10.3760/cma.j.issn.2095-4352.2014.02.009

[22] Malbrain ML Marik PE Witters I Cordemans C Kirkpatrick AW Roberts DJ Van Regenmortel N (2014). Fluid overload, deresuscitation, and outcomes in critically ill or injured patients: a systematic review with suggestions for clinical practice. Anaesthesiol Intensive Ther, 46: 361–380. 25432556 10.5603/AIT.2014.0060

[23] Cordemans C De Laet I Van Regenmortel N Schoonheydt K Dits H Huber W Malbrain ML (2012). Fluid management in critically ill patients: the role of extravascular lung water, abdominal hypertension, capillary leak, and fluid balance. Ann lntensive Care, 2(Suppl 1): S1. 10.1186/2110-5820-2-S1-S1 PMC3390304 22873410

[24] Vaara ST Korhonen AM Kaukonen KM (2012). Fluid overload is associated with an increased risk for 90-day mortality in critically ill patients with renal replacement therapy: data from the prospective FINNAKI study. Crit Care, 16: R197. 23075459 10.1186/cc11682 PMC3682299

[25] ProCESS Investigators Yealy DM Kellum JA (2014). A randomized trial of protocol-based care for early septic shock. N Engl J Med, 370: 1683–93. 24635773 10.1056/NEJMoa1401602 PMC4101700

[26] ANZICS Clinical Trails Group Peake SL Delaney (2014). Goal-Directed Resuscitation for patients with early septic shock. N Engl J Med, 371: 1496–506. 25272316 10.1056/NEJMoa1404380

[27] Singer M (2012). Management of fluid balance: a European perspective. Curr Opin Anaesthesiol, 25: 96–101. 22157192 10.1097/ACO.0b013e32834e8150

[28] Cordemans C De Laet I Van Regenmortel N Schoonheydt K Dits H Martin G Huber W Malbrain ML (2012). Aiming for a negative fluid balance in patients with acute lung injury and increased intra-abdominal pressure: a pilot study looking at the effects of PAL-treatment. Ann Intensive Care, 2(Suppl 1): S15. 22873416 10.1186/2110-5820-2-S1-S15 PMC3390296

